# Manipulation of the Bitcoin market: an agent-based study

**DOI:** 10.1186/s40854-022-00364-3

**Published:** 2022-06-01

**Authors:** Peter Fratrič, Giovanni Sileno, Sander Klous, Tom van Engers

**Affiliations:** 1 Informatics Institute, University of Amsterdam, Amsterdam, The Netherlands; 2 Leibniz Institute, TNO/University of Amsterdam, Amsterdam, The Netherlands

**Keywords:** Agent-based modelling, Cryptocurrency, Market manipulation, Liquidity, Bitcoin

## Abstract

Fraudulent actions of a trader or a group of traders can cause substantial disturbance to the market, both directly influencing the price of an asset or indirectly by misinforming other market participants. Such behavior can be a source of systemic risk and increasing distrust for the market participants, consequences that call for viable countermeasures. Building on the foundations provided by the extant literature, this study aims to design an agent-based market model capable of reproducing the behavior of the Bitcoin market during the time of an alleged Bitcoin price manipulation that occurred between 2017 and early 2018. The model includes the mechanisms of a limit order book market and several agents associated with different trading strategies, including a fraudulent agent, initialized from empirical data and who performs market manipulation. The model is validated with respect to the Bitcoin price as well as the amount of Bitcoins obtained by the fraudulent agent and the traded volume. Simulation results provide a satisfactory fit to historical data. Several price dips and volume anomalies are explained by the actions of the fraudulent trader, completing the known body of evidence extracted from blockchain activity. The model suggests that the presence of the fraudulent agent was essential to obtain Bitcoin price development in the given time period; without this agent, it would have been very unlikely that the price had reached the heights as it did in late 2017. The insights gained from the model, especially the connection between liquidity and manipulation efficiency, unfold a discussion on how to prevent illicit behavior.

## Introduction

Cryptocurrencies are a digital alternative to legal *fiat* money. Rather than being issued by competent governmental authorities, their implementation is based on the principles of cryptography used to validate all transactions and generate new currency. Every transaction that occurs is recorded in a public ledger.^1^

The blockchain, and more in general distributed ledgers, facilitate innovation in multiple domains of activity. These include, but are not limited to, supply chain management, data sharing, accounting, e-voting, or, as the most prominent area, finance [see, e.g., the overview in Casino et al. (2019)]. While it is indisputable that the blockchain by itself had and has a great influence on public discourse, with innovation potential comparable to that of the Internet (as it fosters a decentralized infrastructure for economic transactions), financial experts remain generally skeptical. The implementation and the characteristics (including the strictly technological ones) of blockchain technology, when proposed as a replacement for standard fiat currency are subject to ongoing discussion (Berentsen and Schär 2018; Dierksmeier and Seele 2018; Ertz and Boily 2019; Glaser and Bezzenberger 2015). A major problem surrounding cryptocurrencies—but also, one of the reasons why they have become well known to the general public—are the heavy tails of their return distribution (Chan et al. 2017) and their volatility (Bariviera 2017), resulting in a rich history of “bubbles” (Gerlach et al. 2018).

Although the innovative potential of distributed ledger technologies is vast, the innovation itself does not necessarily translate into trust (see, e.g., Bodó 2021). Traditional markets and exchanges were fairly successful in establishing a trustworthy environment via governmental or international institutions, robust legislative activity, market regulations, and effective monitoring/oversight systems. This development took many decades after a long history of market abuse (Putniņš 2012), and remains an area of active research. It can be said that each new case of market abuse brought a better understanding of market vulnerabilities and often led to viable countermeasures. Furthermore, every new technology potentially brings new techniques for committing fraud. Now, cryptocurrencies, crypto-assets, and various forms of blockchain services are still in their infancy. Therefore, new methods need to be invented or reinvented for this new medium to establish a reliable and fair market environment, ideally while maintaining the decentralized and (semi)anonymous nature of the underlying blockchain technology.

With this motivation, we focus in this study on one example where cryptocurrency market was supposedly manipulated via fraudulent actions of one market participant. A data-driven model is developed and validated using historical data. The behavior of the fraudulent entity is investigated in detail and included in the model. Toward the end, we conclude our investigations with a discussion on how our findings can be applied to improve trust by reducing the present vulnerabilities of crypto-markets. In the remainder of this section, we will provide a brief overview of the study on frauds on cryptocurrencies, on agent-based modeling (especially in the context of crypto markets), and we will then highlight the specific contributions of this paper.

### Fraud and cryptocurrencies

Several illicit activities are related to cryptocurrencies, such as black-market trading (Foley et al. 2019), money laundering, and terrorist financing (Fletcher et al. 2021).^2^ In our case, we focus on fraud that targets and disrupts the market. A more common form of fraud in crypto markets is *wash trading* (Cong et al. 2020; Victor and Weintraud 2021). The principle of wash trading is to execute trades where the buyer and seller are the same entity. Thus, false impressions of highly traded assets are created to mislead investors. Another more serious form of fraud observed in crypto markets is *pump-and-dump* schemes (Kamps and Kleinberg 2018), which typically take the form of coordinated actions to increase the market price in a short time period (Hamrick et al. 2019; Li et al. 2018). In the literature, we find various studies that attempt to explain price as a direct consequence of manipulative behavior. A study (Gandal et al. 2018) analyzed suspicious market practices on the Mt.Cox exchange concludes that fraudulent actions influenced the price growth from $150 to $1000 in late 2013. More recently, Griffin and Shams (2019) argue that the Bitcoin market price might have been inflated by the issuance of Tether.

As observed in a 2014 study (Robleh et al. 2014), Bitcoin and other cryptocurrencies served as a medium of exchange for a relatively small number of people; therefore, they pose no serious material risk to monetary and financial stability, but today investors increasingly involve crypto-assets in their portfolios, and some large companies or payment services are already accepting payments in Bitcoin. This means that cryptocurrency volatility can potentially be a new source of systemic risk to the entire economy and financial sector. Recent studies have approached risk using methods such as clustering (e.g., Li et al. 2021), multi-objective feature selection (e.g., Kou et al. 2021), or network analysis (e.g., Anagnostou et al. 2018). Focusing more on the source of systemic risk originating in illicit behavioral schemes, although advances in *detection* of wash trading (Victor and Weintraud 2021) and pump-and-dump schemes (Chen et al. 2019) are already taking place, new models are needed that can explain, simulate, or possibly predict the effects of fraudulent behavior, and that can serve as a testbed for testing the effectiveness of policies, regulations, or monitoring enforcement mechanisms. One way to satisfy this demand is to consider models that combine qualitative and quantitative knowledge, which can be designed with a strong reliance on empirical data and can simulate various scenarios to address questions regarding the effectiveness of regulatory interventions in the crypto market, as discussed in Shanaev et al. (2020).

### Agent-based modelling

Agent-based models generally aim to explain some complex phenomena, where the emergent behavior at the macro-level is hypothesized to be a consequence of behavioral rules at the micro-level. For a historical review, we refer to Chen (2012). In recent years, this modeling paradigm has been enhanced by more modern data-driven approaches, where behavioral data specific to each agent are used to construct, initialize, or estimate the parameters of a model of each agent’s decision mechanism. Only a relatively small number of parameters are left to be calibrated for the aggregated data, which increases the model’s validity and credibility. With this approach, even large-scale models are capable of rivaling the predictive power of traditional quantitative methods, for example, in the area of economic research (Poledna et al. 2019). These models can be particularly instrumental if the parameters of individual agents are of vital importance, for example, to test interventions during the COVID-19 pandemic (Kerr et al. 2021).

In the literature, several examples of agent-based models can be found that have been created to gain insights into crypto markets. Most of these models are based on various financial or behavioral assumptions. To the best of our knowledge, the first study in this area is Luther (2013), where agents are put into a currency market with switching costs and network effects to investigate the widespread acceptance of cryptocurrency. A similar question was studied by Bornholdt and Sneppen (2014). An implicit assumption of demand was made in Cocco et al. (2017), enhanced by speculative traders and restricted by finite resources for each agent, and is the earliest example of a limit order book-based model of the Bitcoin market attempting to explain the price increase from the start of 2012 to April 2014. This model was later extended by mining (Cocco and Marchesi 2016) and evolutionary computation (Cocco et al. 2019). Other order book models are presented in Pyromallis and Szabo (2019) and Zhou et al. (2017), where the focus is mainly on the adaptive behavior of traders. In Lee et al. (2018), a combination of inverse reinforcement learning directly from Bitcoin blockchain data and order book agent-based modeling was used to make short-term predictions of the market price. Recently, models focusing on policy recommendations have also been developed. Shibano et al. (2020) is introducing a price stabilization agent to reduce the volatility, and Bartolucci et al. (2020) investigates design extension of the Bitcoin blockchain to increase transaction efficiency.

A strong aspect of the agent-based models is that they provide an experimental environment for policymakers. Once a behavioral schema is identified, methods to measure and assess the consequences are settled, and the consequences are measured; the simulated environment can be utilized to test the effectiveness of certain measures, that is, a set of alternative policies to be tested, given some adaptation rate, monitoring, enforcement, and identify the best one. In recent review (Lopez-Rojas and Axelsson 2016) agent-based models are considered as a tool for generating synthetic data for machine learning models, which can be used, for example, to complement more traditional evaluation methods (Kou et al. 2014).

Most notably, agent-based models were developed in the area of urban crime modeling (Groff et al. 2019) or to study the behavioral aspects of tax evasion (Pickhardt and Prinz 2014). In principle, these models are not limited only to observed fraudulent behavior: they can extend the design of fraud committing agents by considering different schemes of market manipulation methods to measure and assess the consequences. By choosing a suitable representation of the fraud schema, it is possible to find more sophisticated patterns of reasoning for a fraudulent agent [e.g., by applying algorithmic evolutionary methods (Hemberg et al. 2016)].

### Contributions

Most studies focus on analyzing the statistical relationship between price and a set of exogenous variables. Conversely, in this study, we focus on the *qualitative* explanation dimension. Our approach builds on the qualitative findings in Griffin and Shams (2019), but, in contrast to this study, we construct a data-driven model, focusing mainly on the causal influence of the fraudulent behavior that supposedly inflated the Bitcoin price. This methodological innovation can be regarded as the main contribution of this study, along with the conceptualization of a specific fraud schema as an algorithm that can be executed by an agent in a simulated cryptocurrency market. Note that this approach opens the door to a broader view on the role of the fraudulent trader in the Bitcoin market, thus allowing to analyze the situation from various points of view. For instance, as our market model is capable of generating market data such as the market price, the market volume or the Bitcoin inflow of the fraudulent trader, it is possible to compare these quantities to empirical data. In particular, we discover that certain anomalies in market volume or dips in market price can be attributed to the actions of a fraudulent trader, an experimental conclusion, which completes the evidence presented in Griffin and Shams (2019).

Furthermore, the model developed in this study allows us to investigate specific reasons behind the success of the market manipulation via the fraud schema. Connections between the efficiency of a specific manipulation strategy and transaction costs^3^ will be explored. To do so: a realistic model of order book liquidity has to be implemented. Most studies implicitly or explicitly assume sufficient liquidity near the mid-price and an exponential decrease in liquidity further away from the mid-price, using a Gaussian assumption, or more relaxed forms.^4^ We propose a new liquidity distribution model based on a mixture of two components. The Gaussian assumption is kept near the mid-price, and beta distribution is used to model the situation more deeply in the order book.

The study of market manipulations (and their consequences) has a long tradition in the economic literature (Putniņš 2012). To the best of our knowledge, the present study is the first to construct an agent that reproduces the actions of a fraudulent trader directly using blockchain transaction data, and reconstructing the market behavior from this predictor. In addition, our simulation environment can be easily expanded with more sophisticated artificial intelligence models, thus contributing to the active area of research concerned by the integration of artificial intelligence with blockchain technology (Pandl et al. 2020; Salah et al. 2019).

Focusing on the economic study dimension of the paper, most of the assumptions we formulate to construct the proposed computational model attempt to provide a sound story (based on previous studies analyzing the Bitcoin market) aiming to reconstruct market behavior in a given time period. Our findings might challenge the opinion that the main predictors of the Bitcoin bubble of late 2017 and the beginning of 2018 would be variables associated with the market sentiment (see Kapar and Olmo 2021). While we do not deny that market sentiment plays a major role, our results confront the thesis that the occurrence of this price bubble is spontaneous or a consequence of the widespread popularity of Bitcoin. In this sense, we contribute to the ongoing discussion among economists on the price formation of cryptocurrencies.

## Background

This section elaborates on the alleged price manipulation using Tether in 2017/18, presenting the technology at stake, the associated socio-technical system, and considerations shared in the relevant literature.

### What is Tether and why is it controversial

Tether is a cryptocurrency whose market price is pegged to the US dollar, making it one of the so-called *stablecoins*. The objective of Tether is to facilitate transactions between cryptocurrency exchanges, making them easier for traders than with fiat money because many exchanges have challenges in establishing banking relationships and meeting their strict regulatory requirements. Tether is issued by *Tether Limited*, which claims that every issued Tether is backed by one dollar. *Tether Limited* publishes end of month (EoM) statements to prove this. This claim is somewhat controversial from several points of view, as discussed in Griffin and Shams (2019), pointing out suspicious auditing methods. Publishing the statement about the reserves potentially gives leverage to the issuer to issue more Tether than the current amount of capital reserves in between the audits. Following a series of investigations started by the New York Attorney General Letitia James filing a suit in April 2019, Bitfinex and Tether agreed to pay a penalty of $18.5 million in a settlement in February 2021. Furthermore, on February 23rd, Attorney General James claimed that Tether had lied about its reserves.^5^

One of the first exchanges to accept Tether, and a close associate to *Tether Limited* by several shareholders, is the Bitfinex exchange. The analysis (Griffin and Shams 2019) exposed and analyzed suspicious flows of Tether from the Bitfinex exchange to other exchanges that accept Tether, mainly Bittrex and Poloniex. Before arriving at the target exchanges, the flow passes through several addresses on the Tether blockchain. Once the Tether is exchanged for Bitcoin, Bitcoin flows back to Bitfinex. As analyzed in their study, these flows were highly correlated with the price increase. Additionally, Griffin and Shams (2019) identified the dominant addresses and concluded that the addresses were likely controlled by the same individual. We will use these insights to model the manipulator’s behavior by observing the change in the balance of the most relevant address.

### Manipulation scheme

**Fig. 1:**
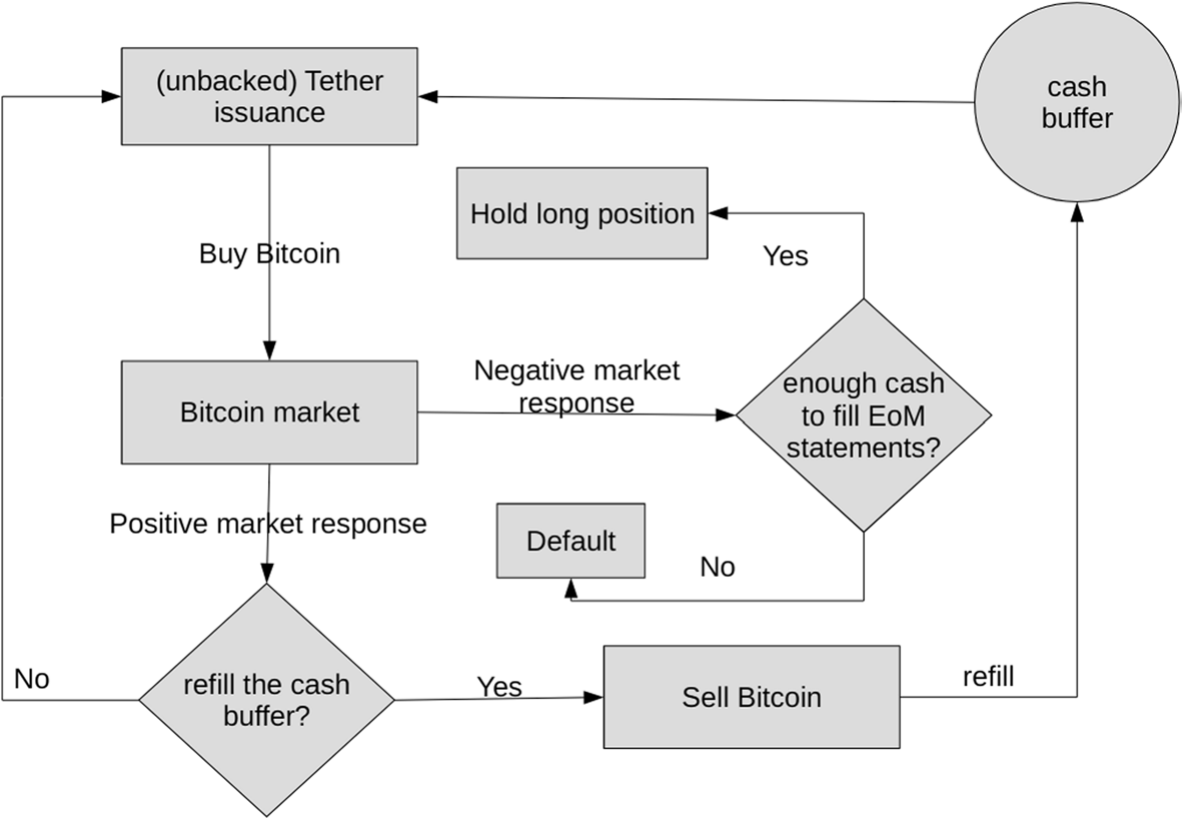
Price inflation scheme. Unbacked Tether is issued and pushed into Bitcoin market. The fraudulent trader must have enough cash to cover the EoM statements

The possibility of pushing Tether into the market gives rise to a simple price inflation scheme that can be placed into the category of *pump-and-dump* schemes. However, as will be explained later, it is even more “powerful” for dimensions in which the profit is generated. In its procedural essence, this scheme can be viewed as an algorithm, and its outline is visualized in Fig. 1 (note that in the real world, many more possibilities of action come into play depending on the circumstances, and the whole scheme can be much more complicated). The strategy of price inflation mostly relies on the assumption that the market will respond with positive feedback (inflow of buy orders) as a consequence of the Bitcoin buy orders executed by the fraudulent trader. Once the positive trend of the market price is established and sustained, the trader”s cash buffer can be refilled if needed, which means that there will be enough cash for the EoM statements to be satisfied. In principle, the positive feedback assumption is unnecessary because a long position is built up even if the market reacts negatively. However, in that case, an additional source of dollars to cover up the EoM statements would be needed; that is, an initial capital or a risk-bearing third party would have to be involved. Then, the trader can sustain the long position and wait until the market conditions are more favorable to restart the scheme.

The profits generated by the scheme in the case of a positive response must be understood in two ways. First, to increase the value of Bitcoins, the fraudulent trader already has possession by triggering the inflow of new buyers. This is the main similarity to the pump-and-dump schemes. Second, as a way to obtain “free” Bitcoin. If the price increased sufficiently, the fraudulent trader would sell smaller amounts of Bitcoins for Dollars than the amount bought with Tether to cover the EoM statements; thus, there will be a surplus of Bitcoins. The crucial question that the fraudulent trader needs to address is deciding on the selling strategy. One plausible strategy would be to pump the price as high as possible and then sell a sufficient amount of Bitcoin by executing a sequence of sell orders a few days before the date of the EoM statement publication. For the reasons explained in later sections, we believe it is cost-effective if the sequence consists of very small sell orders; in this way, the liquidation process takes advantage of high liquidity near the current price, but it can also be harder to notice by the rest of the market participants, and so the price should not drop too drastically. The liquidation strategy via a sequence of small sell orders can be further enhanced by executing small sell orders on multiple exchanges. This would make it more challenging to trace the liquidation process; indeed, though the study of Griffin and Shams (2019) performs an analysis of the outflow from Bitfinex reserves during the times concurrent with the publication of the EoM statements, the question of where these flows end remains unanswered.

### Volume anomalies

In Griffin and Shams (2019), it was concluded that Tether flows from suspicious addresses are correlated with the price increase. We extend these observations in the context of volume and influence on other traders. We argue that, it should be possible to see evidence of fraudulent traders selling their unlawfully obtained Bitcoins in the traded volume to satisfy the EoM statements. Indeed, if the fraudulent trader has an incentive to sell large amounts of Bitcoins within a span of a few days shortly before publishing the EoM statement, or at least somewhere around that time, it is expected that the volume in this time span would temporally increase both directly on the exchanges where the selling takes place and secondarily as a response of other traders reacting to increased amounts of sell orders. In both cases, such actions must be visible in the total Bitcoin trade volume and several large exchanges’ volumes.

*Data collection* As the trade volumes of Poloniex and Bittrex were several times higher trade volumes than other large exchanges such as Coinbase or Bitflyer, we have decided not to use this data, as they probably experienced wash trading. Instead, we used traded volume data from exchanges that obtained a Bitlicense (Chohan 2018) issued by the New York State Department of Financial Services or had similarly reported volumes. We downloaded the volume data from https://data.bitcoinity.org and aggregated the trade volume of trustworthy exchanges (Bitfinex, Bitflyer, Bithumb, Bitstamp, Coinbase, and Kraken) and the total volume of other smaller exchanges.

If Poloniex and Bittrex volumes were not artificially increased, we would naturally use their volumes for model validation; however, this was not the case. For this reason, we need to define what will be our *reference exchange*, which will serve as a reference when analyzing the simulations, to estimate how much influence the fraudulent agent has in terms of traded volume. We then take the volume data of trustworthy exchanges from the same source and take the averages over daily values. As the fraudulent agent was active on two exchanges, we multiply the averages by two.

**Fig. 2:**
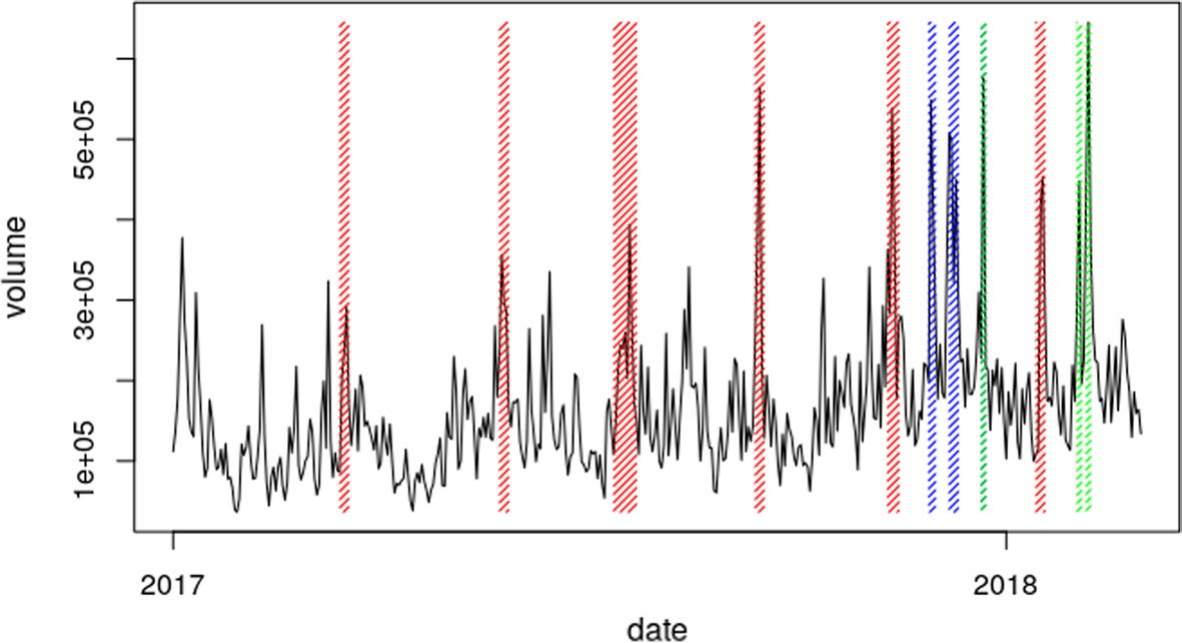
Aggregated volume with highlighted end of month events and large scale events

*Data analysis* Figure 2 reports the resulting aggregated volume. The red bars correspond to the fraudulent agent supposedly liquidating some of the Bitcoins to satisfy the schema in Fig.  1. We will refer to the days when the liquidation process takes place as *EoM events* because the chosen days generally correspond to the end-of-month statements published by *Tether Limited* on the 15th of every month. As the fraudulent trader likely had some initial capital, these days do not have to correspond exactly to the 15th of every month. The general pattern is that these spikes tend to occur every 2 months. As can be seen from Table 1, especially in July, September, November, and January, the liquidation process seems to be matching the 15th day of the month very well.

Additionally, we hypothesize that the blue and green bars in Fig. 2 correspond to the market responding to an increase or decrease in price as a consequence of actions performed by the fraudulent trader. The blue bars correspond to the volume increase due to an increase in buying, and the green bars correspond to an increase in selling. We refer to these days as *large scale events (LSE)*. A possible explanation for these events is that some investors entering or leaving the market temporally increased the volume, triggering a secondary response from other traders. However, the true reason behind these volume anomalies remains an open question. Given the uncertainty and as this study aims to focus on the modeling of a fraudulent trader, we will not attempt to model LSEs as actions of some specific agents, but we will assume them in the simulation as prior knowledge (exogenous events).

### Inter-exchange influence and liquidity

Before we start building the agent-based model of the market, it is important to discuss our assumption that influencing the price on two exchanges is sufficient to influence the market price across all other exchanges. The direct way in which one exchange can influence the price is by trading large volumes of Bitcoin. Most web services that report the price of Bitcoin calculate the price as an average over the last traded price on several exchanges, weighted by the traded volume. These services must have a way of detecting wash trading, but they can hardly filter out a fraudulent trade, such as the one described in previous sections. Therefore, if seemingly legal fraudulent trades of large volumes are executed on one exchange, then the reported price will be skewed by the activity of this exchange, diminishing the influence of the other exchanges. It is clear that if fraudulent buy orders are matched with sell orders with high limit prices, the calculated Bitcoin market price will consequently be pushed higher than the average price traded on other exchanges.

A second way the activity on one exchange can influence the whole market is by traders observing price fluctuations on multiple exchanges and generating a profit by taking advantage of these small price differences. It was concluded in Chordia et al. (2008) that such an *arbitrage* activity, if stimulated by sufficient liquidity, results in higher price efficiency, which, in turn, results in a more stable market price unless new external information enters the market. However, in Marshall et al. (2018), analyzing a database of Bitcoin intraday data on 14 exchanges, including prices of 13 currencies, it was observed that cryptocurrency markets tend to be illiquid and hence less price-efficient. This means that there is a lower overall agreement on the price of Bitcoin. From this, it can be concluded that the variations in price across all major exchanges, given the low liquidity of Bitcoin, can increase price volatility. Indeed, in the same study, evidence shows that an increase in illiquidity corresponds with an increase in crash risk across all pairs when liquidity proxies are either the effective spread or price impact. This volatility–liquidity relationship was confirmed by some studies (Næs and Skjeltorp 2006; Tripathi et al. 2020; Valenzuela et al. 2015) from a quantitative point of view.

Based on this argument, one might expect ascendancy among different cryptocurrency exchanges. The earliest study to investigate this question is Brandvold et al. (2015). This study discusses a leader–follower relationship between various exchanges, linking them to specific events regarding Chinese government policies or the arrest of the Silk Road black market owner (October 2, 2013). Interestingly, the Mt. Gox exchange was identified to have a large but decreasing information share in the market; however, during the period concurrent with the price manipulation period described in Gandal et al. (2018), the Mt. Gox exchange again established its dominant position in the market. This is not only consistent with previous arguments and provides an early example that manipulative behavior on one exchange can influence the price of the entire market.

In conclusion, illiquidity and low agreement among traders about the price of Bitcoin create favorable conditions for a manipulation scheme to be executed successfully. In later sections, we extend the discussion on illiquidity in greater detail, showing that the way liquidity is distributed in the order book can provide an essential advantage for the fraudulent trader.

## Exchange model

The level of granularity assumed for our investigation is a *limit order book* model in which orders are placed in a public order book. An order can be entered every second in the order book in cryptocurrency exchanges. In our exchange model, the orders can enter every minute to simplify processing, which means that each trading day *d* consists of $T=1440$ tics (minutes). We use the time index *t* to measure the time in the model in minutes, and we use the time index $\tau$ to measure the time in days; for example, $p_{t}$ denotes the price at time *t*, and $p_{\tau }$ denotes the price at the end of a trading day $\tau$.

### Limit order book market model

The market environment is based on the model presented in Raberto et al. (2005). Each trader can observe the order book $O_t$ at time *t*; that is, a table consisting of 5 columns: order type, Bitcoin amount, residual amount, limit price, issue day, and expiration day. With respect to the limit price, the buy orders are sorted in descending order and the sell orders are sorted in ascending order. Issue time is the second sorting criterion when the limit prices are equal. Each trading day is split into *T* tics during which traders can issue orders. If the issue day exceeds the expiration day, the order is removed from the order book. Market orders^6^ by setting the limit price to zero. At the time *t*, we denote $B_j[O_t]$ as the limit price of the *j*-th buy order, and $S_i[O_t]$ as the limit price of the *i*-th sell order. The sell order of index *i* and the buy order of index *j* are matched if and only if $S_i[O_t] \le B_j[O_t]$. The order-matching mechanism is defined as follows:

- if $$S_i[O_t] = 0$$ or $$B_j[O_t] = 0$$:
  - if $$B_j[O_t] > 0$$, then $$p_{t} \xleftarrow []{} min(B_j[O_t],p_{t})$$
  - if $$S_j[O_t] > 0$$, then $$p_{t} \xleftarrow []{} max(S_j[O_t],p_{t})$$
- if $S_i[O_t] = 0$ and $B_j[O_t] = 0$, then $p_{t} \xleftarrow []{} p_{t}$
- if $S_i[O_t] > 0$ and $B_j[O_t] > 0$, then $p_{t} \xleftarrow []{} \frac{B_j[O_t] + S_j[O_t]}{2}$

Every time a new order enters the order book, the first sell and buy orders are inspected if they satisfy $S_i[O_t] \le B_j[O_t]$, and the new market price is decided according to the order-matching mechanism. As more than one order can be issued at time *t*, the last match at time *t* is the current price $p_{t}$. We do not consider expiration times within a minute during the simulation because this would unnecessarily complicate the model.

### Expiration time, price and amount distributions

One factor that determines the price and crucial property of every exchange is the *order book depth*. In principle, the order book depth is defined by the distribution of Bitcoin amounts and the limit prices placed in the order book by traders. In our environment, almost all traders decide the Bitcoin amount and limit price by sampling these two values from predefined distributions, thus filling the order book with orders.

Based on the findings presented in Schnaubelt et al. (2019), we hypothesize that four main empirical properties are relevant to our study.

1. broad hump-shaped (bimodal) distribution of limit prices;
2. quickly rising transaction costs;
3. relatively small volume concentrated around the mid-price, compared to total volume provided by the order book;
4. both sides of the order book are on average symmetric with respect to the mid-price.

We assume that the limit price and Bitcoin amount distributions are independent for simplicity. We assume that the bimodal shape of the limit price distribution is due to a mixture of two distributions. The first component is modeled by a Gaussian distribution $N(\mu ,\sigma )$, with mean $\mu$ and variance $\sigma$. The second component, representing the tail of the limit price distribution, is modeled by a beta distribution $Beta(\alpha ,\beta )$, where $\alpha ,\beta$ are the shape parameters. To produce an on average symmetric distribution, the limit price in the former case is defined as $p_t \cdot N(\mu ,\sigma )$ for buy orders and $\frac{p_t}{N(\mu ,\sigma )}$ for sell orders. For the tail, we must introduce two additional parameters *a*, *c*: the location parameter *a* and the scale parameter *c* (Johnson et al. 1995). Then, the limit price of orders placed deeper into the order book is for buy orders:

1a $$\begin{aligned} LimitPriceTailBuy_t \sim p_t[1+c + (c-a)Beta(a,b)] \end{aligned}$$

and for sell orders:

1b $$\begin{aligned} LimitPriceTailSell_t \sim p_t[1-c + (c-a)Beta(a,b)] \end{aligned}$$

The second component defining market depth is the *amount distribution*. As we mainly control the transaction costs using the limit prices, the amount distribution is less important, but we will attempt to make it realistic nonetheless. Several characteristic properties of the amount distribution were observed empirically (Cong et al. 2020). The main characteristic to be captured is the bias of traders to certain “round” values, such as $0.5,1,1.5,2,\dots$. We construct this distribution as a mixed discrete/continuous distribution consisting of a Poisson distribution and an exponential distribution of the form:

2 $$\begin{aligned} Amounts \sim (1-q)(0.5+0.5\cdot Pois(\lambda _P)) + q \cdot Exp(\lambda _E) \end{aligned}$$

where $q \in [0,1]$ and $\lambda _P,\lambda _E$ are rate parameters.

Finally, the *expiration time* of an order influences the distribution of limit prices and amounts over time. Similar to Cocco et al. (2017), we use the floor value of the log-normal distribution with the parameters $\mu _{L},\sigma _L$. In the simulation, we set these parameters to relatively low values because it seems plausible to assume that traders will be cautious in keeping any order in the order book for too long, given the uncertainty about the Bitcoin price. In addition, we assume independence between the expiration time conditioned on price and amount.

## Agent models

The success of a scheme used by the fraudulent trader depends on the response of the market. Therefore, we discuss the *market response model* or *market response agents* when referring to the response of the market to the actions of the *fraudulent agent* (FA).

### Market response agents

*Random agents* Random agents (RAs) are issuing buy or sell orders with equal probability and hold with probability $1-P_{RA}$. The limit price is sampled from the Gaussian component defined above.

*Random speculative agents* Random speculative agents (RSAs) are issuing buy or sell orders the same way as RAs. The limit price is sampled from the Beta distribution according to the Eqs. (1a) and (1b), which means the limit prices of their orders are relatively far away from the mid-price. Therefore, the RSA speculates that even orders placed deeper in the order book will be matched given the market’s volatility. The probability that the RSA will hold is $1-P_{RSA}$.

*Chartist agents* Chartist agents (CAs) are observing the average of Bitcoin returns in the window $$[\tau -l,\tau ]$$ over which the average is taken. The probability that a CA will issue order is $$P_{CA}$$. If the average return is positive, the CA issues a buy order; otherwise, a sell order. The limit price is sampled from the Gaussian component. CAs are active if the market price is above $50, and they follow their initial strategy until the price reaches $20000. Subsequently, the CA will decide with probability $$Q_{CA}$$ to issue a sell order and with probability $$(1-Q_{CA})P_{CA}$$ to continue the initial trend-following strategy. Parameter $$Q_{CA}$$ can be interpreted as the CA belief that the price will drop after reaching its presumed maximum. If the price happens to decrease to $10000, the CA will return to a pure trend following [for this threshold price approach, see, for instance, Lee and Lee (2021)].

### Fraudulent agent

In principle, the fraudulent agent behavioral script is defined by the buying and selling schedules. The buying schedule is constructed directly from the available data on Tether outflows. The selling schedule is constructed following the discussion in previous sections, considering the empirical findings related to Bitcoin order book liquidity.

*Cash matrix* A cash matrix *C*(*t*) defines the amount of cash that the FA will use to issue a buy order on a given day and minute. Using this capital, the FA calculates the amount of Bitcoins to buy from the order book and then issues a market order. Let us define $b_t$ as the amount of Bitcoin the FA has in possession at time *t*. The amount of Bitcoin to be obtained at $b_{t+1}$ depends on the available cash allocated in the cash matrix and the state of the order book. The cash matrix was constructed from the amounts of Tether sent from the 1J1d and 1AA6 addresses, as identified in Griffin and Shams (2019), spanning 1 year and 3 months from January 1, 2017, to March 1, 2018. Ninety percent of Tether flows from Bitfinex to Poloniex go to the 1J1d deposit address, and 72% of Tether flows from Bitfinex to Bittrex go to 1AA6.^7^ If we identify one Tether with one USD, ignoring negligible fluctuations in the price of Tether, then these flows provide a compelling picture of the FA’s capital. As the timescale of the model is minutes per day, the Tether flows are aggregated per minute. As the market model is a scaled-down model of an exchange, the cash matrix also needs to be scaled down, which is done by multiplying the cash matrix element-wise with the scalar parameter *s*.

*Selling strategy* The selling strategy is a strategy of the FA to liquidate a portion of the Bitcoins to refill the cash buffer and then satisfy the EoM statements. We claim that these selling days roughly correspond to the date when EoM statements are published by *Tether Limited*, which is the 15th of every month, but the FA does not need to meet this deadline strictly, given that the FA most likely have backup capital available. Although there are no strict consequences for the FA for not fulfilling the obligations in the model environment, we assume that if $b_t < 0$ at any point in time, the FA will exit the market to maintain a long position on the obtained Bitcoins. The exit of the FA typically occurs when the market response is not sufficiently positive, and the price is too low for the FA to regain capital by selling Bitcoins.

If everything goes as planned, the FA will sell a small amount of Bitcoins every minute by issuing a limit sell order, decreasing the number of Bitcoins $b_t$ that the FA has in possession at time *t*. As the order book is relatively liquid near the mid-price, it is logical for the FA to issue only small sell orders and avoid large sell orders because of the rapid increase in transaction costs. Thus, the FA aims to obtain a fraction $\frac{c_i}{1440}$ of the total cash that was used to obtain Bitcoins, where $c_i$ are the coefficients in Table 1, telling us how much of the cash is planned to be obtained on a specific day. The coefficients are calculated from empirical data by taking the values of the traded volume and dividing each value by a normalizing constant. For instance, if the traded volume on September 14 was 484601.8 Bitcoins and September 15 was 705641.0 Bitcoins, to obtain the coefficients, each value is divided by the sum $484601.8 + 705641.0$; thus, $0.4071453 + 0.5928547 = 1$. This means that on September 14, the FA plans to obtain $40.7\%$ and the following day $59.3\%$ of the capital deficit present in the cash buffer.

**Table 1:** List of EoM events and amount of cash planned to obtain at given day in order to cover the expenses incurred by buying Bitcoin

Year	Month	Day	Fraction of total expenses to regain
2017	March	18th	1.0000000
2017	May	25th	0.5440995
		26th	0.4559005
2017	July	14th	0.1362958
		15th	0.1945955
		16th	0.2200540
		17th	0.2153293
		18th	0.2337253
2017	September	14th	0.4071453
		15th	0.5928547
2017	November	10th	0.5612051
		11th	0.4387949
2018	January	14th	0.3628918
		15th	0.3922153
		16th	0.2448929

### Large scale events

Volume anomalies that do not seem to be related to the actions of the FA are regarded as LSEs. While it might be possible to model these spikes in traded volume as actions of certain types of agents, we take an easier path of using the information present in the traded volume data.

The dates in which LSEs occurred are extracted from Fig. 2 and listed in Table 2, together with a hypothesis on whether an LSE consisted of predominantly buy or sell orders, which is not possible to read from volume data alone, but can be assumed depending on the trend in the market price.^8^ This means that, in addition to standard trading activity during one day, an increase in trading activity is arranged by issuing more orders to reproduce the green and blue volume anomalies in Fig. 2. The magnitude of an LSE is defined by the number of orders issued on a given day, and the amount of Bitcoin bought or sold per order. As we do not have data records related to LSE events, we make the simplifying assumption that the orders during one LSE day arrive with a frequency *f* to trade amount $\rho$; that is, every *f* minutes a new market order is issued to buy or sell $\rho$ Bitcoins. Additionally, depending on the exact date, the amount $\rho$ is multiplied by a scaling factor such that the volume anomaly during the simulation matches the empirical volume anomaly. The scaling factors are listed in Table  2.

**Table 2:** List of large scale events associated with volume spikes, that are not explained by EoM events

Year	Month	Day	LSE scaling factor	Order type
2017	November	29th	3.538633	Buy
2017	November	30th	2.651711	Buy
2017	December	7th	3.275189	Buy
2017	December	8th	3.216667	Buy
2017	December	9th	2.076381	Buy
2017	December	10th	2.900676	Sell
2017	December	11th	1.965138	Buy
2018	January	22nd	3.716152	Sell
2018	February	1st	1.901109	Sell
2018	February	5th	2.866988	Sell
2018	February	6th	4.163546	Sell

## Experiments and results

To demonstrate the essential influence of FA on the market, four simulation experiments are presented:

1. *Non-manipulated scenarios:*
  - *Base scenario*
  - *Susceptible scenario*
  - *Susceptible scenario with large scale events*
2. *Manipulated scenario*.

Thus, the market price time series can be decomposed in terms of activity of agents. To ensure that the results are consistent in all three scenarios:, the model parameters are kept the same as listed in Table 3, except for setting parameters defining the activity of excluded agents or events zero for each of the first three scenarios. In non-manipulated scenarios, the market price time series is the central quantity that provides information on the behavior of the underlying system. In the manipulated scenario, three more quantities related to the activity and influence of the FA are measured along with the price. These quantities are:

- The *Market Price* generated by the model is compared to the Bitcoin market price.
- The *Volume* generated by the model is compared to the reference exchange as defined in the section on volume anomalies. Both empirical and simulated volumes were normalized for comparison on the same scale.
- The *Inflow of Bitcoin* as obtained by the FA during the simulation is compared to the inflow of Bitcoin to the 1LSg address. As in the case of volume, both the empirical and simulated inflows were normalized.
- The *Relative Influence* of the FA is defined as the ratio of Inflow of Bitcoin and the Volume. In this case, normalization is not needed.

**Table 3:** Parameters of the model

Description of the parameter	Symbol	Value
Number of simulations	*n*	100
Number of days	*N*	425
Number of tics	*T*	1440
Mean value Gaussian (limit price) distribution	$\mu$	1
Variance of the Gaussian (limit price) distribution	$\sigma$	0.1125
1st shape parameter of the Beta (limit price) distribution	$\alpha$	2.85
2st shape parameter of the Beta (limit price) distribution	$\beta$	1
Location parameter of the Beta (limit price) distribution	*a*	0.015
Scale parameter of the Beta (limit price) distribution	*c*	0.5
Mixture weight of the amount distribution	*q*	0.05
Rate parameter of the Poisson distribution	$\lambda _{P}$	2.0
Rate parameter of the Exponential distribution	$\lambda _{E}$	1.0
Location parameter of the log-normal distribution	$\mu _L$	1.5
Shape parameter of the log-normal distribution	$\sigma _L$	0.1
Probability of the RA to issue an order	$P_{RA}$	0.2583
Probability of the RSA to issue an order	$P_{RSA}$	0.15
Probability of the CA to issue an order	$P_{CA}$	0.03625
Belief of the CA that the price will drop	$Q_{CA}$	0.0085
Size of the window of the returns	*l*	6
Cash matrix scale parameter	*s*	0.00172
LSE amount	$\rho$	0.45
Intraday frequency of LSE orders	*f*	25 min

Empirical data from January 1, 2017, to March 1, 2018, are used to calibrate the model parameters, and the results are visualized for each scenario (Abel 2015). Some parameters in Table 3 were predefined based on empirical findings (see the “Discussion” section), and the rest of the parameters were calibrated using * stochastic simultaneous optimistic optimization* algorithm (Valko et al. 2013), except for parameter *l*, which was calibrated manually. More details about the calibration can be found in the “Appendix”.

**Fig. 3:**
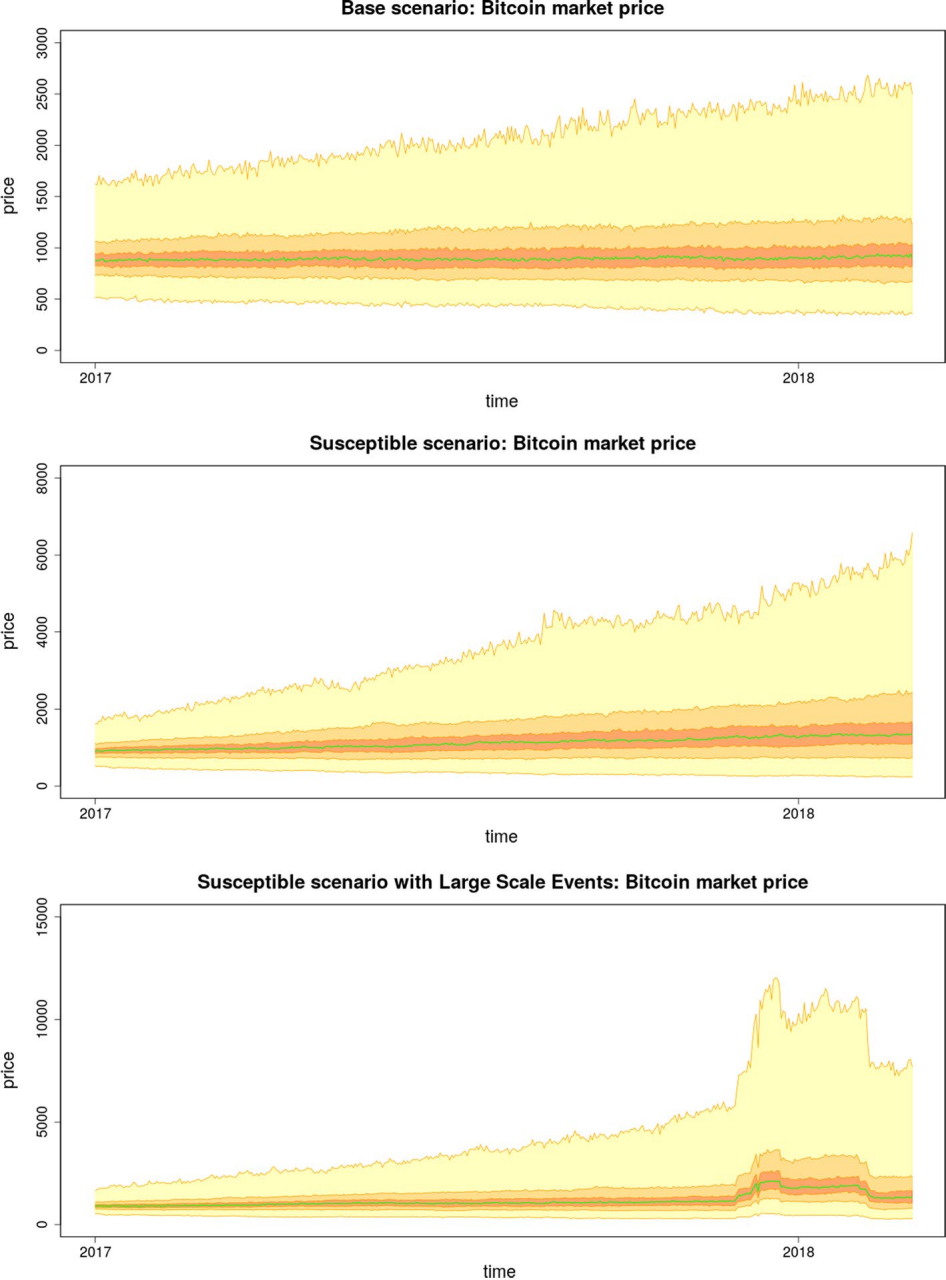
Simulated market price time series in terms of activity of agents or presence of large scale events. Base scenario with only random agents and random speculative agents; susceptible scenario including Chartist agents; and susceptible scenario with large scale events included in the simulation. The green line is the median price with 20th, 50th and 95th prediction interval

### Non-manipulated scenarios

In the base scenario, we set $$\rho = s = P_{CA} = 0$$, which means that the FA and CAs are not active, and the scaling factor of the additional amounts bought or sold during the LSEs is multiplied by zero. In the susceptible scenario, the CAs are active and issue orders with a given probability. We refer to this scenario as “susceptible” because, contrary to the base scenario, the market with CAs is prone to large price fluctuations. However, as will be apparent from the simulations, even if LSEs are included, the probability of a price reaching $20000 is rather unlikely.

**Fig. 4:**
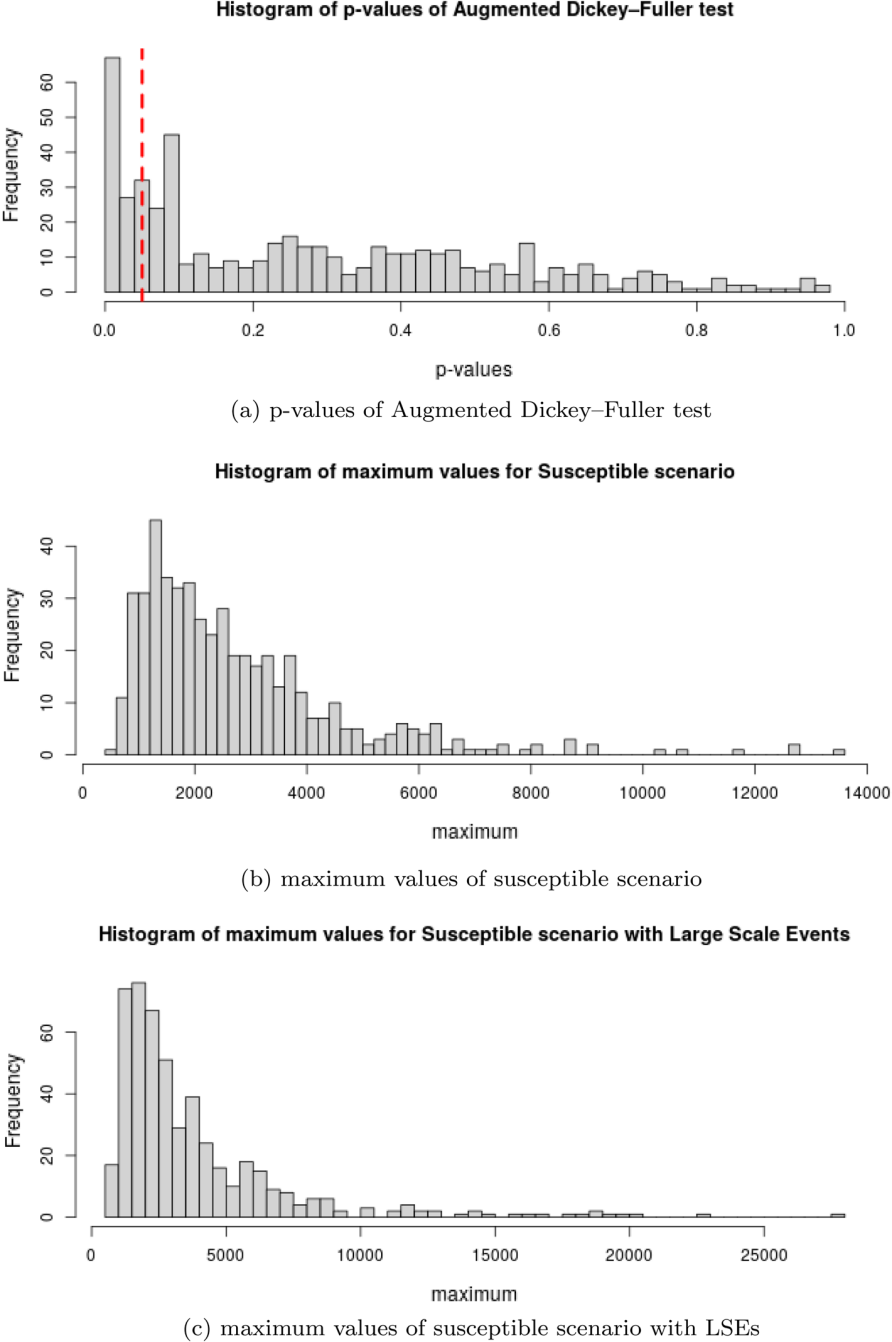
Histograms related to non-manipulated scenarios. In subfigure (**a**) the histogram of p-values of Augmented Dickey-Fuller test calculated for each simulation of the base scenario is plotted with a red dashed line at value 0.05. In subfigures (**b**) and (**c**) the histograms of maximum values of the market price achieved during each simulation are plotted for susceptible scenario and susceptible scenario with LSEs, respectively

#### Base scenario

This is a scenario where the market is in an equilibrium state, which is intuitive to be expected because, with no speculation present on the market and a sufficient amount of liquidity on both sides of the order book, a large fluctuation in the price is improbable to occur. By calculating the *p* value of the augmented Dickey–Fuller test for stationarity for each simulation of the base scenario, we obtain a distribution of *p* values, as depicted in Fig. 4a. From this histogram, we can see that the alternative hypothesis of stationarity dominates.

#### Susceptible scenario

This scenario includes agents that are following the trend, and therefore one can expect larger price fluctuations to be observed. However, although in this case, the stationarity test did not provide evidence for stationarity, the price time series is considerably “well-behaved.” Indeed, if we look at the histogram of the maximum values (Fig. 4b), there is only a minimal number of simulations that are capable of surpassing the $10000 Bitcoin price.

#### Susceptible scenario with large scale events

This scenario includes both the speculative behavior of the CAs and disturbances in the form of LSEs. As shown in Fig. 3, the mean value of the price temporarily shifts before the LSE sells orders to lower the price to its long-term value. Overall, this disturbance is insufficient to produce an increasing trend, even when CAs are present.

### Manipulated scenario

**Fig. 5:**
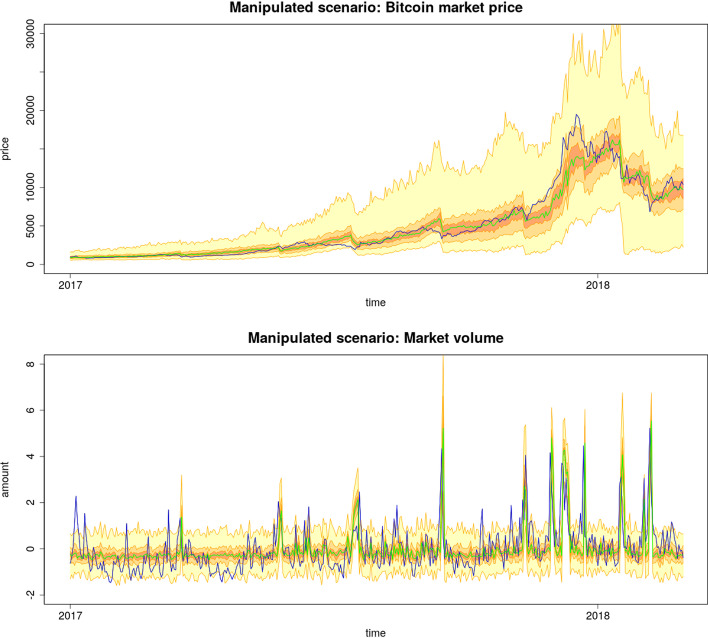
Simulated market price and market volume with Fraudulent agent included during the simulation, along with the large scale events and all the agents of the response model. The empirical data (blue) are plotted against simulated median (green) with 20th, 50th and 95th prediction interval

In this scenario, the FA is active during the simulation, and all parameters are set as shown in Table 3. In Fig. 5, we can see the consequences of the presence of FA compared with the non-manipulated scenarios visualized in Fig. 3. The influence of EoM events is visible on the price time series and, together with LSEs, form spikes in the volume. Typically, the FA decides to hold a long position in 20–25% of the cases. The trajectories of these unfinished manipulation attempts are excluded from the figures because they represent a different market regime that needs a different dataset to be validated.

**Fig. 6:**
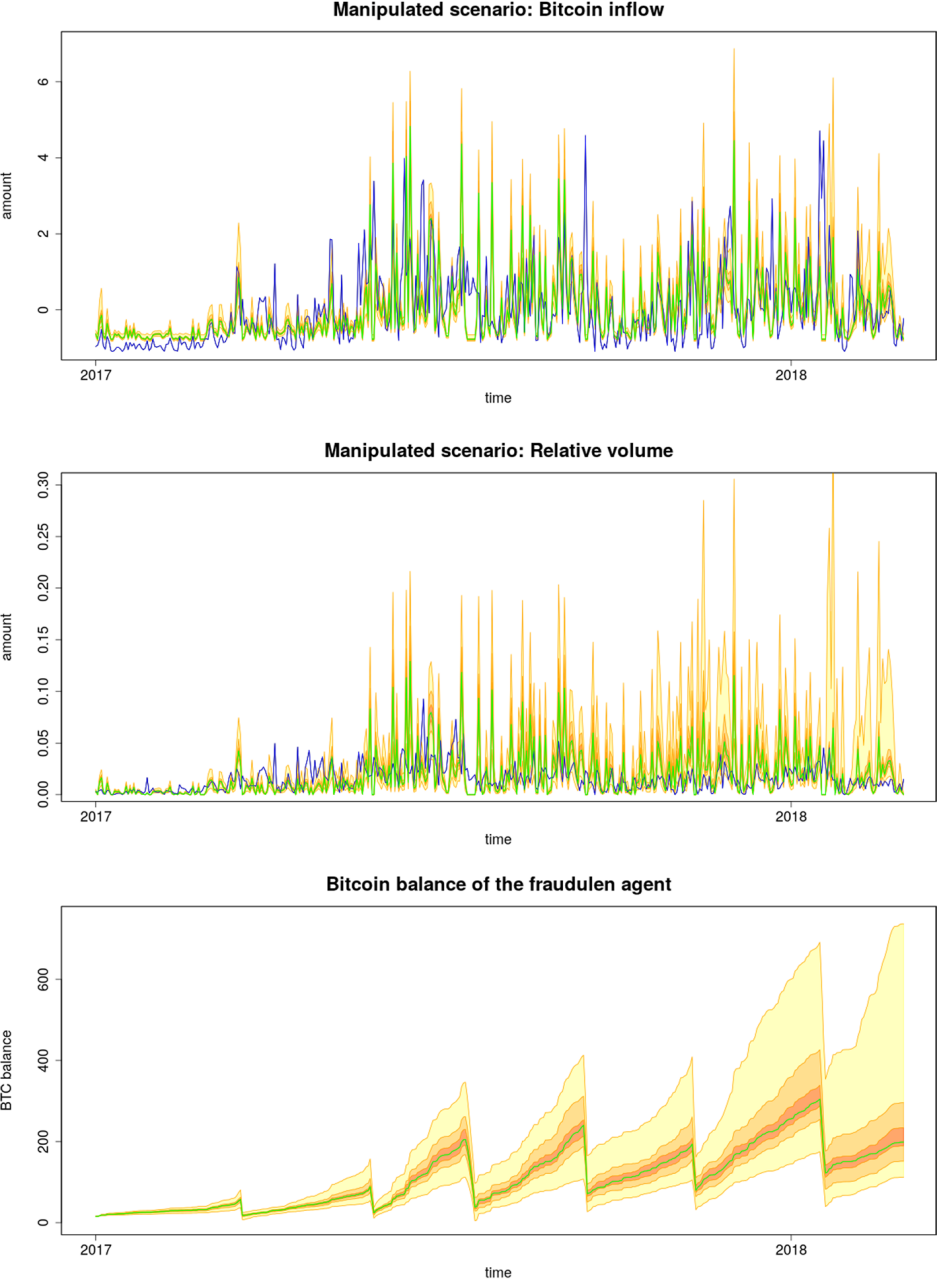
Time series detailing the behavior of the fraudulent agent with respect to empirical data (blue); compared to the simulated median (green) with 20th, 50th and 95th prediction interval

If everything goes as planned, the FA buys Bitcoins using allocated cash in the cash matrix, as shown in Fig. 6, where simulated Bitcoin inflows measured in the model are plotted against the inflow of Bitcoin into the 1LSg address. It can be seen that the Tether outflow encoded in the cash matrix is produced via the market simulation with almost the same Bitcoin inflow as that obtained from the real Bitcoin blockchain. By aggregating these simulated daily inflows, the Bitcoin balance $b_t$ is obtained and displayed in Fig. 6, where sudden drops owing to EoM events are visible. The balance increases approximately linearly between the drops, and a surplus of Bitcoin is produced over a longer period. Note that the surplus was produced only by executing Scheme 1, and no resources (Tether or Dollar) were spent. In other words, other market participants paid a bill.

### Limit order book market robustness

The liquidity of the order book is a strong predictor of the success of a scheme defined by Fig. 1. Increasing liquidity by increasing the number of orders issued by random agents using parameters $P_{RA}$ and $P_{RSA}$, or by increasing the amounts issued per order using parameters of the amount distribution, would be the most straightforward way to make the order book more liquid. In this case, assuming the FA would not adapt, the relative influence of the FA would decrease; thus, the market would be more resistant to manipulation attempts.

**Fig. 7:**
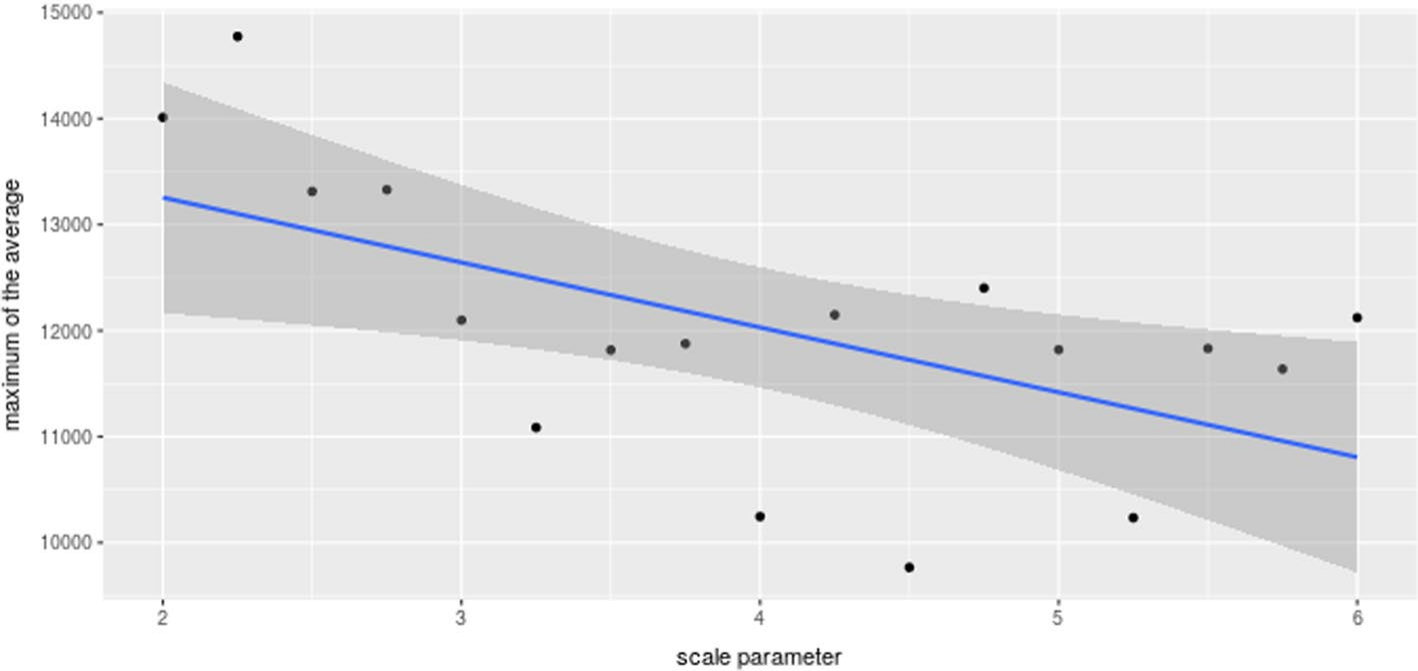
The maximal value of price time series averaged over 80 simulations is plotted against the parameter $\alpha$ of the Beta distribution controlling the liquidity deeper in the order book

What is perhaps less obvious is that not only the total amount of liquidity, but also the distribution of liquidity is a relevant factor. As noted previously, traders’ low agreement about the price of an asset is translated into the dispersion of the limit prices further away from the mid-price. Indeed, if traders agreed on the asset’s market price, they would put their orders much closer to the mid-price. More orders concentrated closer to the mid-price would result in lower transaction costs; therefore, the efficiency of the FA’s manipulation strategy should be lower.

This hypothesis can easily be tested in our model environment. By increasing the parameter $\alpha$, the orders with limit prices previously placed further away from the mid-price will now be placed closer to the mid-price because increasing the first shape parameter of the beta distribution, while keeping the second shape parameter equal to one, will move the mass of the density function toward the value of its location parameter *a*. This means that there are more orders with a limit price close to $(1+a)p_{t}$ for sell orders and close to $(1-a)p_{t}$ for buy orders. As shown in Fig. 7, by the increasing parameter $\alpha$, the efficiency of the manipulation strategy decreases because the inflated price decreases.

The consequence of the FA is that, despite buying more Bitcoin for the same amount of Tether, the price impact is lower because the FA’s buy orders do not match sell orders with as high limit prices, thus, changing the distribution of liquidity, in our case, by controlling the parameter $\alpha$, has a similar effect as increasing the overall liquidity. Note that the parameter $\alpha$ has little effect during EoM events because the FA sells Bitcoin in small amounts, matching buy orders near the mid-price.

As the FA has a virtually unlimited amount of Tether to push into the Bitcoin market, it is possible to issue more Tether. However, this would increase the risk associated with the given manipulation scheme; thus, the fraudulent trader would need to increase the backup capital or default in case of insufficiently positive market response. Indeed, by increasing the parameter $\alpha$ in our computational experiment, the number of FA defaults was higher. Furthermore, note that even if the FA manages successfully to execute the scheme, the profits would be lower, while the risk would increase.

## Discussion

### Methodological concerns

In the present work, the design of the model follows an *incremental strategy*, increasing the complexity until a sufficiently good fit to the empirical data is obtained.^9^ This approach is well suited to this case study because the essential importance of the FA was demonstrated by decomposing the market price time series in terms of agents” activities. Given the high level of consistency of our assumptions with other empirical studies found in the economic literature and the satisfactory fit to empirical data related to the Bitcoin market, high confidence can be given to the modeling assumptions related to the principles behind the success of the manipulation scheme investigated in this study.

Some of the parameter values in Table 3 were set to match the empirical observations of Bitcoin limit-order books (Schnaubelt et al. 2019). It was observed that orders are placed as far as $50\%$ from the mid-price, so we set $c=0.5$. The location of the local maximum in the hump-shaped average order book was observed to be approximately $1\%$ from the mid-price. This fact is also reflected in the model by setting $a=0.015$. The parameters of the amount distribution (2) were similarly predefined, considering the findings in Cong et al. (2020). The calibration results agree with known empirical observations. As the RA issuing an order is higher than the probability of the RSA, most of the liquidity will be located near the mid-price. However, due to the relatively low value of the $\alpha$ parameter, it is still possible to observe orders further away from the mid-price, which is again in agreement with the findings in Schnaubelt et al. (2019).

Although the model implements several realistic assumptions, many simplifications cause higher prediction errors. For instance, for the reasons described in the section discussing volume anomalies we deem a plausible assumption, that it was sufficient for the fraudulent trader to influence the price on Poloniex and Bittrex, which means that to model a manipulation on the entire Bitcoin market, it should be sufficient to model the manipulation using only one order book. However, such a simplification is not sufficient to fully consider EoM events. If the FA can liquidate Bitcoins on multiple exchanges in small amounts, then this process is more price-efficient than liquidating on a single exchange. This means that the influence of the real fraudulent trader could have been even slightly higher, and thus the parameter *s* is probably underestimated.

The simulated data did not produce very good results, especially from the end of May until the end of July, roughly between the 2nd and 3rd EoM events. The activity of chartist traders likely depends on the average returns and the Bitcoin market value, which means that the CA ought to be less active if the price is low. This is not the case in the model because parameter $P_{CA}$ is constant. Moreover, to obtain a better fit for the empirical data, it would be necessary to include the flows from the dominant Tether addresses and the flows from all Tether addresses controlled by the fraudulent trader. It is also possible that the fraudulent trader followed a less aggressive selling strategy prior to the third EoM event and started the liquidation process before July 14, 2017. In the fragrant Bitcoin market, it is challenging to correctly identify the reasons behind some of the insufficiencies present in our model because even actions with negligible influence on the price in more liquid markets can significantly influence illiquid Bitcoin market.

### Regulatory implications

The economic understanding going with the proposed model has important implications for the contemporary cryptocurrency market. A regulation where stablecoin providers must prove their capital not only once a month but in a much shorter time period is highly desirable to protect—the customers of these providers and other participants in the market—from being misled into a pump-and-dump scheme. Policymakers are slowly catching up with the industry in terms of legislative regulation. The European Union Commission proposed and agreed on a legal framework for cryptocurrencies,^10^ especially targeting stablecoins in their “Regulation on Markets in Crypto Assets” proposal. In the U.S., President Biden’s administration has also recently taken a proactive stand on stablecoin regulation.^11^

Individual governments can decide the strength of regulations in agreement to their long-term strategy and consider the consequences of their decisions concerning innovation. These decisions can be effectively implemented at the domestic level; however, there might be an incentive to avoid regulations in the case of exchanges, as they can pose the risk of a decrease in traded volume or engage in illicit behavior. In addition to the legislative regulations implemented in various countries, a different self-regulatory approach can be adopted. Regulations to protect the stability of a market by restricting trading mechanisms are already in place on FOREX markets, for instance, constraints on the maximum amount issued by one order, a maximum number of orders of a trader per day, or maximum limit price. Some of these simple restrictions have already been implemented on more regulated exchanges, such as Huobi or Coinbase. Another more invasive intervention is circuit breakers such as price limits or trading halts (Sifat and Mohamad 2019). These regulations would make it more challenging to facilitate manipulative activities but might be perceived as too restrictive, slowing down the sector’s growth. Following the discussion on Bitcoin limit order book market robustness, we can target a dynamic approach to prevent market manipulation without affecting daily trade traffic. Having a better understanding of how liquidity is linked to market manipulation, an exchange can implement a market surveillance system (Cumming and Johan 2008) to inspect liquidity distribution in real-time and predict the market impact of an issued order (Gu et al. 2008; Weber and Rosenow 2005). Then, the exchange can refuse to accept an order if there is suspicion that the order aims to create a sudden increase or decrease in the market price. Moreover, exchanges can search for fraudulent behavioral trading patterns in the order books, directly on the blockchain, in aggregated statistics, or even on public forums, and then evaluate the risk of the trading behavior being associated with fraudulent activity and either intervene by refusing to accept orders or report the suspicion to a relevant authority. As identified in this study, the typical (volume) pattern of Scheme 1 is manifested in approximately periodic spikes in the traded volume. A well-designed monitoring system should be capable of detecting suspicious addresses that repeatedly issue buy orders with a relatively high predicted market impact on a few specific exchanges, followed by high Bitcoin liquidation in roughly periodic intervals on some different exchanges, thus probably engaging in the execution of Scheme 1. It is likely that if such a monitoring system were implemented, the manipulation following Scheme 1 would be ineffective.

The advantage of the approach described above is that, on the blockchain, all transactions are public and immutable. Any monitoring system can access the full transaction history, which is usually not the case in traditional finance. This property offers, in principle innovation potential for sophisticated self-learning AI models to oversee market behavior. These models can be trained on historical datasets or simulated environments capable of reproducing fraudulent patterns, such as those presented in this study. However, one must be aware of the possible limitations that often arise from the adversarial nature of these systems. Therefore, present detection tools, therefore, might not be powerful enough to deal with more sophisticated fraud schemes, and more studies need to be done in this area.

While it is true that the clear benefit for the exchanges in implementing regulatory systems to reduce or inhibit market manipulation would stabilize the market, this might be challenging to achieve without an overarching authority. Moreover, as to a certain extent exchanges benefit of fraudulent behavior, there might not be enough incentives to combat fraud: the short-term benefits of the current state of affairs may be more appealing than the long-term benefits of a reliable medium of exchange. For instance, in Kim et al. (2021), the effectiveness of money laundering reporting through exchanges is questioned. This study assumes that exchanges benefit from money laundering; reporting suspicious transactions can increase money laundering activity. One must be aware that a similar situation can occur when dealing with market manipulation. It can be argued that one of the main reasons for the widespread popularization of Bitcoin was the price increase orchestrated in 2017. Even though the exchanges likely knew about the issue,^12^ as apparent both from the statistical evidence presented in Griffin and Shams (2019) and EoM events reconstruction by our model, the manipulation continued.

## Conclusion and further research directions

It was demonstrated that introducing a fraudulent agent with a price manipulation strategy could create a price bubble that would not occur or would occur only with practically zero probability. The model can also explain several quantitative phenomena. Most anomalies, such as dips in the market price or spikes in the market volume during 2017 and the beginning of 2018, were connected to the end-of-month statements of *Tether Limited*. We hypothesize that the remaining anomalies can be explained by the inflow of new investors in response to the positive trend in market price due to price manipulation. Additionally, the efficiency of a price manipulation scheme was connected to several studies on order book liquidity and price formation. Dependency on the shape of the liquidity distribution is discussed and demonstrated computationally.

The results of our model provide important insights to further the understanding of exchange manipulation with possible impacts on the entire market. These findings can be fruitful for policymakers and regulators when designing suitable countermeasures against market abuse. In addition, the proposed countermeasures can be tested in a simulated environment, such as the one presented in this study or one similar to ours, going in the promising direction of deep integration of distributed ledger technologies and artificial intelligence. These research directions may be closely related to study-contingent economic arrangements or experimental financial instruments. Should a decentralized monetary system work; it seems essential to implement a set of regulations that prevent manipulation attempts, or at least make it more challenging to apply them successfully.

This model can be extended in several ways. The two most obvious extensions are to use full information from the addresses related to the market manipulator, as in Griffin and Shams (2019), or to use detailed order book data, as in Schnaubelt et al. (2019), but directly for the exchanges involved. Combining the datasets of these two studies with our model can potentially remove some of the remaining misalignments and provide a better fit for market price, relative volume, and realized inflow. Furthermore, a more sophisticated approach can be adopted when designing the fraudulent agent and the response agents, a choice that would include more complex behavioral rules and allow the agents to be active on several exchanges. In particular, the fraudulent trader should be enabled to observe and act upon the liquidity situation in the order book, the response of the market, and the possible market abuse countermeasures that may be included in the simulated environment. Finally, if a sufficiently rich market model is attained, the knowledge and understanding obtained by analyzing its function can be used to update the trading infrastructure of Bitcoin. The methodology developed in this research area has the potential to be further generalized and applied to another novel economic and financial infrastructures.

## Data Availability

Source code of the model can be found at https://github.com/fratric/Bitcoin-Price-Manipulation. All data were downloaded from public sources. Price and volume data can be found at the gitHub repository, together with processed (Tether and Bitcoin) blockchain data, that were obtained using blockchain-explorer application programming interface available at https://bitcoinchain.com and https://blockchain.com.

## Appendix: simulation and calibration details

In principle, we are interested to find such values of model parameters that provide a good fit to the price time series and do not overestimate the influence of exogenous elements such as the activity of FA or the magnitude of LSEs. This means that the accuracy of the model needs to be defined either as a multi-objective function, or as a single-objective function that sums weighted components of the multi-objective functions, where: the first component measures the error between generated and empirical market price; the second component measures the error between generated and empirical market volume; the third component measures the error between generated and empirical relative volume.

In the optimization routine we choose the simpler weighted option. Furthermore, if the empirical and generated market volume is standardized, then the volume peaks already provide an information about the influence of the FA (through EoM events) and the influence of the LSEs both relative to the spikes in empirical volume. This means that by measuring the distance between the generated and the empirical volume during the EoM or LSE days, we already impose a penalty if the algorithm would decide to overestimate the influence of the EoM and LSE related parameters, namely *s* and $\rho$. Therefore the objective function measuring the accuracy of the model for the parameter vector $\theta = (\sigma , \alpha , P_{CA}, Q_{CA}, P_{RA}, P_{RSA}, s, \rho )$ with predefined values for $\mu , \beta , a, c, q, \lambda _P, \lambda _E, l$ can be simplified to:

3 $$\begin{aligned} Err(\theta ) = \frac{1}{N} \sum _{\tau =1}^{N} |p_{\tau } - {\tilde{p}}_{\tau }| + w \max _{\tau \in D} |v_{\tau } - {\tilde{u}}_{\tau }| \end{aligned}$$

This provides a compromise between complexity and accuracy. The weight is $w = 400$. The symbols ${\tilde{p}}_{\tau }$ and ${\tilde{u}}_{\tau }$ denote the median time series taken over a collection of 16 trajectories of generated price and volume respectively, in order to counter the stochasticity of the model output.

Most of the parameters of the model are relatively sensitive and since the response model agents do not have bounds on available capital, certain parameter configurations can cause the market price to grow exponentially, or to decline basically to zero. This extreme behavior mainly depends on the value of the parameter *l*. The parameter *l* and the bounds on the parameter vector $\theta$ were decided during the initial exploration of the simulation output. The bounds on the parameter $\theta$ are listed in table 4.

**Table 4:** Parameter bounds used during the stochastic simultaneous optimistic optimization algorithm

	$\sigma$	$\alpha$	$P_{CA}$	$Q_{CA}$	$P_{RA}$	$P_{RSA}$	*s*	$\rho$
Lower bound	0.05	2.10	0.035	0.007	0.25	0.1	0.001	0.375
Upper bound	0.125	3.0	0.0425	0.01	0.30	0.2	0.002	0.525

## Abbreviations

FA: Fraudulent agent
CA: Chartist agent
RA: Random agent
RSA: Random speculative agent
LSE: Large scale event
EoM: End of month
AI: Artificial intelligence

## Footnotes

1 For a short introduction to the most known cryptocurrency, the Bitcoin, we refer to Böhme et al. (2015) and for an overview on others we refer to Berentsen and Schär (2018).

2 For a review and more examples, we refer to Badawi and Jourdan (2020).

3 Defined as the premium a trader has to pay to liquidate a given amount of assets.

4 For example in Raberto et al. (2005) a Gaussian assumption is employed, which is also used in cryptocurrency setting (Cocco and Marchesi 2016; Cocco et al. 2017, 2019). Several studies relaxed the Gaussian assumption with either a log-normal assumption (Bartolozzi 2010), or a power-law assumption (Cui and Brabazon 2012; McGroarty et al. 2019).

5 The final decision was published as a press release on https://ag.ny.gov/press-release/2021/attorney-general-james-ends-virtual-currency-trading-platform-bitfinexs-illegal.

6 A marker order is a buy order to buy for the best sell limit price, or a sell order to sell for the best buy limit price.

7 We have obtained data related to these addresses using the API available at https://omniexplorer.info.

8 In contrast, in the case of EoM events, the increase in sell orders is due to the manipulation scheme 1.

9 Also known as TAPAS (Take A Previous model and Add Something) strategy.

10 See the press release https://www.consilium.europa.eu/en/press/press-releases/2021/11/24/digital-finance-package-council-reaches-agreement-on-mica-and-dora/ and related documents.

11 Press release https://home.treasury.gov/news/press-releases/jy0454 of the U.S. Department of the Treasury.

12 First series of blogposts occurred in August 2017 on https://bitfinexed.medium.com/ with a coverage in The New York Times https://www.nytimes.com/2017/11/21/technology/bitcoin-bitfinex-tether.html by the end of November 2017.

## References

[CR1] Abel GJ (2015). fanplot: an r package for visualising sequential distributions. R J.

[CR2] Anagnostou I, Sourabh S, Kandhai D (2018). Incorporating contagion in portfolio credit risk models using network theory. Complexity.

[CR3] Badawi E, Jourdan G-V (2020). Cryptocurrencies emerging threats and defensive mechanisms: a systematic literature review. IEEE Access.

[CR4] Bariviera AF (2017) The inefficiency of Bitcoin revisited: a dynamic approach. Econ Lett 161(2017):1–4. 10.1016/j.econlet.2017.09.013. arXiv:1709.08090

[CR5] Bartolozzi M (2010). A multi agent model for the limit order book dynamics. Eur Phys J B.

[CR6] Bartolucci S, Caccioli F, Vivo P (2020). A percolation model for the emergence of the Bitcoin Lightning Network. Sci Rep.

[CR7] Berentsen A, Schär F (2018). A short introduction to the world of cryptocurrencies. Fed Reserve Bank St. Louis Rev.

[CR8] Bodó B (2021). Mediated trust: a theoretical framework to address the trustworthiness of technological trust mediators. New Media Soc.

[CR9] Böhme R, Christin N, Edelman B, Moore T (2015). Bitcoin: economics, technology, and governance. J Econ Perspect.

[CR10] Bornholdt S, Sneppen K (2014) Do Bitcoins make the world go round? On the dynamics of competing crypto-currencies, pp 1–5. arXiv:1403.6378

[CR11] Brandvold M, Molnár P, Vagstad K, Christian O, Valstad A (2015). Price discovery on Bitcoin exchanges. J Int Financ Mark Inst Money.

[CR12] Casino F, Dasaklis TK, Patsakis C (2019). A systematic literature review of blockchain-based applications: current status, classification and open issues. Telemat Inform.

[CR13] Chan S, Chu J, Nadarajah S, Osterrieder J (2017). A statistical analysis of cryptocurrencies. J Risk Financ Manag.

[CR14] Chen S-H (2012). Varieties of agents in agent-based computational economics: a historical and an interdisciplinary perspective. J Econ Dyn Control.

[CR15] Chen W, Xu Y, Zheng Z, Zhou Y, Yang JE, Bian J (2019) Detecting ‘Pump & dump schemes’ on cryptocurrency market using an improved a priori algorithm. In: Proceedings—13th IEEE international conference on service-oriented system engineering, SOSE 2019, 10th international workshop on joint cloud computing, JCC 2019 and 2019 IEEE international workshop on cloud computing in robotic systems, CCRS 2019, pp 293–298. 10.1109/SOSE.2019.00050

[CR16] Chohan U (2018). Oversight and regulation of cryptocurrencies: BitLicense. SSRN Electron J.

[CR17] Chordia T, Roll R, Subrahmanyam A (2008). Liquidity and market efficiency. J Financ Econ.

[CR18] Cocco L, Marchesi M (2016) Modeling and simulation of the economics of mining in the Bitcoin market. PLoS ONE 11(10):1–31. 10.1371/journal.pone.0164603. arXiv:1605.01354 PMC5074464 27768691

[CR19] Cocco L, Concas G, Marchesi M (2017) Using an artificial financial market for studying a cryptocurrency market. J Econ Interact Coord 12(2):345–365. 10.1007/s11403-015-0168-2. arXiv:1406.6496

[CR20] Cocco L, Tonelli R, Marchesi M (2019). An agent-based artificial market model for studying the Bitcoin trading. IEEE Access.

[CR21] Cong L, Li X, Tang K, Yang Y (2020). Crypto wash trading. SSRN Electron J.

[CR22] Cui W, Brabazon A (2012) An agent-based modeling approach to study price impact. In: 2012 IEEE Conference on computational intelligence for financial engineering economics (CIFEr), pp 1–8. 10.1109/CIFEr.2012.6327798

[CR23] Cumming D, Johan S (2008). Global market surveillance. Am Law Econ Rev.

[CR24] Dierksmeier C, Seele P (2018). Cryptocurrencies and business ethics. J Bus Ethics.

[CR25] Ertz M, Boily É (2019). The rise of the digital economy: thoughts on blockchain technology and cryptocurrencies for the collaborative economy. Int J Innov Stud.

[CR26] Fletcher E, Larkin C, Corbet S (2021). Countering money laundering and terrorist financing: a case for Bitcoin regulation. Res Int Bus Finance.

[CR27] Foley S, Karlsen JR, Putnins TJ (2019). Sex, drugs, and Bitcoin: how much illegal activity is financed through cryptocurrencies?. Rev Financ Stud.

[CR28] Gandal N, Hamrick J, Moore T, Oberman T (2018). Price manipulation in the Bitcoin ecosystem. J Monet Econ.

[CR29] Gerlach JC, Demos G, Sornette D (2018) Dissection of Bitcoin’s multiscale bubble history from January 2012 to February 2018, 1–42 (February 2018). arXiv:1804.06261 10.1098/rsos.180643 PMC6689597 31417685

[CR30] Glaser F, Bezzenberger L (2015) Beyond cryptocurrencies—a taxonomy of decentralized consensus systems. In: 23rd European conference on information systems (ECIS), Münster, Germany

[CR31] Griffin JM, Shams A (2019). Is Bitcoin really untethered?. J Finance.

[CR32] Groff ER, Johnson SD, Thornton A (2019). State of the art in agent-based modeling of urban crime: an overview. J Quantit Criminol.

[CR33] Gu G-F, Chen W, Zhou W-X (2008) Empirical shape function of limit-order books in the Chinese stock market. Phys A Stat Mech Appl 387(21):5182–5188. 10.1016/j.physa.2008.05.008. arXiv:0801.3712

[CR34] Hamrick J, Rouhi F, Mukherjee A, Feder A, Gandal N, Moore T, Vasek M (2019). The economics of cryptocurrency pump and dump schemes. SSRN Electron J.

[CR35] Hemberg E, Rosen J, Warner G, Wijesinghe S, O’Reilly U-M (2016). Detecting tax evasion: a co-evolutionary approach. Artif Intell Law.

[CR36] Johnson NL, Kotz S, Balakrishnan N (1995). Continuous univariate distributions.

[CR37] Kamps J, Kleinberg B (2018). To the moon: defining and detecting cryptocurrency pump-and-dumps. Crime Sci.

[CR38] Kapar B, Olmo J (2021). Analysis of Bitcoin prices using market and sentiment variables. World Econ.

[CR39] Kerr CC, Stuart RM, Mistry D, Abeysuriya RG, Rosenfeld K, Hart GR, Núñez RC, Cohen JA, Selvaraj P, Hagedorn B, George L, Jastrzȩbski M, Izzo AS, Fowler G, Palmer A, Delport D, Scott N, Kelly SL, Bennette CS, Wagner BG, Chang ST, Oron AP, Wenger EA, Panovska-Griffiths J, Famulare M, Klein DJ (2021). Covasim: an agent-based model of COVID-19 dynamics and interventions. PLoS Comput Biol.

[CR40] Kim D, Bilgin MH, Ryu D (2021). Are suspicious activity reporting requirements for cryptocurrency exchanges effective?. Financ Innov.

[CR41] Kou G, Peng Y, Wang G (2014). Evaluation of clustering algorithms for financial risk analysis using MCDM methods. Inf Sci.

[CR42] Kou G, Xu Y, Peng Y, Shen F, Chen Y, Chang K, Kou S (2021). Bankruptcy prediction for SMEs using transactional data and two-stage multiobjective feature selection. Decis Supp Syst.

[CR43] Lee S, Lee K (2021). 3% Rules the market: herding behavior of a group of investors, asset market volatility, and return to the group in an agent-based model. J Econ Interact Coord.

[CR44] Lee K, Ulkuatam S, Beling P, Scherer W (2018). Generating synthetic Bitcoin transactions and predicting market price movement via inverse reinforcement learning and agent-based modeling. JASSS.

[CR45] Li T, Shin D, Wang B (2018). Cryptocurrency pump-and-dump schemes. SSRN Electron J.

[CR46] Li T, Kou G, Peng Y, Yu PS (2021). An integrated cluster detection, optimization, and interpretation approach for financial data. IEEE Trans Cybern.

[CR47] Lopez-Rojas EA, Axelsson S (2016) A review of computer simulation for fraud detection research in financial datasets. In: 2016 Future technologies conference (FTC), pp 932–935

[CR48] Luther WJ (2013). Crypto-currencies, network effects, and switching costs. SSRN Electron J.

[CR49] Marshall BR, Nguyen NH, Visaltanachoti N (2018). Bitcoin liquidity. SSRN Electron J.

[CR50] McGroarty F, Booth A, Gerding E, Chinthalapati VLR (2019). High frequency trading strategies, market fragility and price spikes: an agent based model perspective. Ann Oper Res.

[CR51] Næs R, Skjeltorp JA (2006). Order book characteristics and the volume–volatility relation: empirical evidence from a limit order market. J Financ Mark.

[CR52] Pandl KD, Thiebes S, Schmidt-Kraepelin M, Sunyaev A (2020). On the convergence of artificial intelligence and distributed ledger technology: a scoping review and future research agenda. IEEE Access.

[CR53] Pickhardt M, Prinz A (2014). Behavioral dynamics of tax evasion—a survey. J Econ Psychol.

[CR54] Poledna S, Miess MG, Hommes CH (2019). Economic forecasting with an agent-based model. SSRN Electron J.

[CR55] Putniņš TJ (2012). Market manipulation: a survey. J Econ Surv.

[CR56] Pyromallis C, Szabo C (2019) Modelling and analysis of adaptability and emergent behavior in a cryptocurrency market. In: 2019 IEEE Symposium series on computational intelligence, SSCI 2019, pp 284–292. 10.1109/SSCI44817.2019.9002829

[CR57] Raberto M, Cincotti S, Dose C, Focardi SM, Marchesi M, Lux T, Samanidou E, Reitz S (2005). Price formation in an artificial market: limit order book versus matching of supply and demand. Nonlinear dynamics and heterogeneous interacting agents.

[CR58] Robleh A, John B, Roger C, James S (2014). The economics of digital currencies. Bank Engl Q Bull 2014.

[CR59] Salah K, Rehman MHU, Nizamuddin N, Al-Fuqaha A (2019). Blockchain for AI: review and open research challenges. IEEE Access.

[CR60] Schnaubelt M, Rende J, Krauss C (2019). Testing stylized facts of Bitcoin limit order books. J Risk Financ Manag.

[CR61] Shanaev S, Sharma S, Ghimire B, Shuraeva A (2020). Taming the blockchain beast? Regulatory implications for the cryptocurrency market. Res Int Bus Finance.

[CR62] Shibano K, Lin R, Mogi G (2020) Volatility reducing effect by introducing a price stabilization agent on cryptocurrencies trading. In: ACM International conference proceeding series, pp 85–89. 10.1145/3390566.3391679

[CR63] Sifat IM, Mohamad A (2019). Circuit breakers as market stability levers: a survey of research, praxis, and challenges. Int J Finance Econ.

[CR64] Tripathi A, Dixit A, Vipul V (2020). Liquidity of financial markets: a review. Stud Econ Finance.

[CR65] Valenzuela M, Zer I, Fryzlewicz P, Rheinländer T (2015). Relative liquidity and future volatility. J Financ Mark.

[CR66] Valko M, Carpentier A, Munos R (2013) Stochastic simultaneous optimistic optimization. In: International conference on machine learning. PMLR, pp 19–27

[CR67] Victor F, Weintraud AM (2021) Detecting and quantifying wash trading on decentralized cryptocurrency exchanges. In: The web conference 2021—proceedings of the world wide web conference, WWW 2021 2, pp 23–32. 10.1145/3442381.3449824. arXiv:2102.07001

[CR68] Weber P, Rosenow B (2005). Order book approach to price impact. Quantit Finance.

[CR69] Zhou Q, Zhang Q, Zhang Q (2017). Agent-based simulation research on Bitcoin price fluctuation. DEStech Trans Comput Sci Eng (AIEA).

